# The effect of erector spinae plane block on fentanyl consumption during open abdominal hysterectomy: a randomised controlled study

**DOI:** 10.1186/s12871-023-02156-3

**Published:** 2023-06-05

**Authors:** Mohamed Ahmed Hamed, Maged Labib Boules, Mohamed Abd El Moniem Mahmoud, Rana Ahmed Abdelghaffar

**Affiliations:** Department of Anesthesiology, Faculty of Medicine, Fayoum University, Fayoum, 63511 Egypt

**Keywords:** Fentanyl consumption, Postoperative pain, Abdominal hysterectomy, Erector spinae plane block

## Abstract

**Background:**

Perioperative analgesia is very important during an abdominal hysterectomy. Determining the impact of the erector spinae plane block (ESPB) on patients undergoing an open abdominal hysterectomy while under general anesthesia was our aim.

**Methods:**

In order to create equal groups, 100 patients who underwent elective open abdominal hysterectomies under general anesthesia were enlisted. The preoperative bilateral ESPB with 20 ml of bupivacaine 0.25% was administered to the ESPB group (*n* = 50). The same procedure was performed on the control group (*n* = 50), but they received a 20-ml saline injection instead. The primary outcome is the total amount of fentanyl consumed during surgery.

**Results:**

We found that the mean (SD) intraoperative fentanyl consumption was significantly lower in the ESPB group than in the control group (82.9 (27.4) g vs. 148.5 (44.8) g, with a 95% CI = -80.3 to -50.8; *p* 0.001). Likewise, mean (SD) postoperative fentanyl consumption was significantly lower in the ESPB group than in the control group (442.4 (17.8) g vs. 477.9 (10.4) g, with a 95% CI = -41.3 to -29.7; *p* 0.001). On the other hand, there is no statistically significant difference between the two study groups regarding sevoflurane consumption (89.2 (19.5) ml vs. 92.4 (15.3) ml, with a 95% CI = -10.1 to 3.8; *p* 0.4).

We documented that during the post-operative period (0–24 h), VAS scores at rest were, on average, 1.03 units lower in the ESPB group (estimate = -1.03, 95% CI = -1.16-(-0.86), t = -14.9, *p*-value 0.001), and VAS scores during cough were, on average, 1.07 units lower in the ESPB group (estimate = -1.07, 95% CI = -1.21-(-0.93), t = -14.8, *p*-value 0.001).

**Conclusion:**

Bilateral ESPB can be utilized as an adjuvant method to reduce intraoperative fentanyl consumption and enhance postoperative pain control in patients undergoing open total abdominal hysterectomy under general anesthesia. It is effective, secure, and little obtrusive.

**Trial registration:**

No protocol revisions or study amendments have been made since the trial's inception, according to the information on ClinicalTrials.gov (NCT05072184; principal investigator: Mohamed Ahmed Hamed; date of registration: October 28, 2021).

## Background

In order to increase patient comfort, enable early mobilization, lower the risk of thromboembolism, and reduce the length of time patients must stay in the hospital after an abdominal hysterectomy, it is crucial to provide patients with adequate perioperative analgesia [1].

Opioid-based analgesia is the standard of care, but it may have undesirable side effects, such as postoperative nausea and vomiting, itching, constipation, or even potentially fatal respiratory depression [2, 3].

Despite being routinely used during abdominal hysterectomy, epidural anesthesia is limited in patients who have coagulopathy, local infections, or increased intracranial pressure [4].

Forero et al. first described the erector spinae plane block (ESPB) in 2016 [5]. This interfacial plane block is utilized to offer postoperative analgesia, particularly in abdominal and thoracic surgery.

Recent studies have demonstrated that bilateral ESP blocks, when given at the low thoracic level during abdominal surgeries including abdominal hysterectomy [6, 7] and caesarean Sects. [8, 9], produced adequate analgesia.To perform this block, a local anaesthetic is administered under ultrasound guidance between the erector spinae muscle and the transverse processes of the vertebrae. The dorsal and ventral roots of the thoracic and abdominal spinal nerves are subsequently blocked [10].

In this clinical trial, it was supposed that bilateral ESPB would lessen the need for intraoperative fentanyl during open abdominal hysterectomy. Therefore, our goal was to assess how ESPB affected individuals having an open abdominal hysterectomy while under general anesthesia. Intraoperative fentanyl use was the main study outcome.

## Methods

According to the principles of the Declaration of Helsinki, this prospective clinical trial was conducted as a randomized, triple blinded, parallel-group study. The study at Fayoum University Hospital (M546) received approval from the ethical review board, and participants' written agreement was obtained. After being registered on ClinicalTrials.gov (NCT05072184; primary investigator: Mohamed Ahmed Hamed; date of registration: October 28, 2021; no study alterations or protocol amendments after trial commencement), the study was conducted at Fayoum University Hospital from October 2021 to July 2022 as part of this clinical trial. The applicable consortium recommendations were followed in this study.

One hundred individuals who were over the age of 18 and had physical status I or II according to the American Society of Anesthesiologists (ASA) were included in the study.

The following participants were excluded: patients who refused enrollment; significant kidney, liver, and cardiovascular conditions; a history of drug allergy under study; whenever there is a regional anaesthetic contraindication, such as a localized infection or bleeding issues; persistent opioid use; chronic pain history; and cognitive issues.

Randomly, the patients were assigned to two groups: Preoperatively, under US guidance, the ESPB group (50) underwent bilateral ESPB with 20 ml of bupivacaine 0.25%. During the same procedure, 20 ml of saline were administered in a 1:1 ratio to the control group (50) using a computer-generated random table. The patient's assigned group information was taken from a sealed, opaque packet by the anesthesiologist who performed the ESPB but was not involved in any other data collection or patient care. The teams responsible for patient care and data collection, the surgical and anaesthetic teams, and the patients were blinded to the group assignments.

### Anesthesia and block procedure

Granisetron and dexamethasone were used to premedicate all patients prior to surgery. Using noninvasive methods such noninvasive electrocardiography, pulse oximetry, capnography, temperature monitoring, and bispectral index (BIS), all patients were observed in the operating room. A 22G, 50 mm block needle (SONOTAP, Pajunk, Geisingen, Germany) was inserted sterilely at a 30°–40° angle from cranial to caudal into the plane between the fascia of the ESM and the transverse process. The right needle tip position was confirmed by visualizing the linear fluid spread separating the transverse process from the erector spinae muscle. 20 cc of 0.25% bupivacaine was then injected deeply into the erector spinae muscle. On the other side, the same procedure was used. The control group had the same technique but received a sham injection instead (20 ml of saline).

After the ESPB, the patient received a standard anaesthetic induction using intravenous fentanyl (1 μg /kg) and propofol (2 mg/kg). Before tracheal intubation and the mechanical ventilator were employed to maintain the end-tidal CO2 between 30 and 35 mmHg, atracurium (0.5 mg/kg) was given. Depending on the requirements of the patient, both inhaled (Sevoflurane) and intravenous (IV) atracurium were utilized for anaesthetic maintenance.

Sevoflurane was used to sustain the anesthesia at a flow rate of 3 L/min in a 50% oxygen/air combination. Sevoflurane was administered initially at a concentration of 2%, and the concentration was increased to achieve an appropriate level of anesthesia by titrating the dose in accordance with the BIS monitoring (BIS Complete Monitoring System P/N 185–0151 Covidien IIc, 15 Hampshire Street, Mansfield, MA 02048 USA) to maintain the BIS value between 40 and 60. Inadequate (or excessive) sedation was treated by increasing (or decreasing) the concentration of sevoflurane by 0.4 units until the desired effect was reached.

Blood pressure was assessed every five minutes. If the SBP was less than 90 mmHg and the BIS readings were between 40 and 60, a repeat bolus of 5 mg ephedrine was intravenously administered. If the SBP was less than 90 mmHg and the BIS value was less than 40, the sevoflurane vaporizer was decreased by 0.4% until a BIS value of 40 or higher was attained. The sevoflurane vaporizer was increased by 0.4% until a BIS value of 60 or less was attained. When the BIS value exceeded 60 and the SBP was more than 165 mmHg, Assuming that inadequate analgesia was the primary cause of hypertension, a bolus of 1 μg /kg of fentanyl was administered until the SBP was satisfactory if the SBP was higher than 165 mmHg and the BIS values were between 40 and 60. If the heart rate dropped below 50 beats per minute during the operation, 1 mg of atropine was administered intravenously.

After the surgery, sevoflurane was terminated, and neostigmine (0.04 mg/kg) and atropine (0.01 mg/kg) were used for the reversal of muscle relaxation. Once spontaneous breathing started again, the patient was extubated.

The total sevoflurane consumption was calculated according to the formula previously described by Biro [11].

At the end of the operation, all patients were checked into the Post-Anesthesia Care Unit (PACU). With a 10 µg/ml concentration, a 25 g bolus, a 15-min lockout period, and a 100 µg/hr maximum dose with no background dose, all patients received IV fentanyl via patient-controlled analgesia (PCA). In addition, 1 g of IV paracetamol was administered every 8 h. The patient's pain was measured using a 10-cm Visual Analogue Scale (VAS) in the PACU immediately following surgery, as well as at 4, 8, 12, 16, and 24 h later within the ward.

### Measured parameters

The total intra-operative fentanyl consumption (µg) was the primary outcome. The use of intraoperative sevoflurane (ml), postoperative fentanyl in the first 24 h (µg), systolic blood pressure, the mean VAS score at rest and with cough (score 0 = no pain and 10 = worst pain ever), any complications involving the block, general anesthesia, and IV opioids were considered secondary outcomes.

### Statistical analysis and sample size estimation

The sample size was calculated using G-Power software version 3.1.7 (Institute of Experimental Psychology, Heinrich Heine University, Dusseldorf, Germany). A minimum sample size of fifty patients was required for each group. Based on results from other studies [12], the type I error was 0.05 with two tails, the power was 80%, and the effect size was 0.55.

To enable data manipulation, data was gathered, coded, and double-entered into Microsoft Access. The Statistical Package of Social Science (SPSS) software version 22 running on Windows 7 was used to analyze the data (SPSS Inc., Chicago, IL, USA). Simple descriptive analysis using percentages and figures for qualitative data, mathematical means or medians for measuring central tendency, and standard deviations or IQRs for quantifying dispersion For numerical data, the quantitative measures between the two independent groups were compared using independent sample t-tests or Mann–Whitney tests.

The Chi-square test was used on qualitative data to compare two or more qualitative groups. With a *P* value of 0.05, it was judged statistically significant.

## Results

**Fig. 1:**
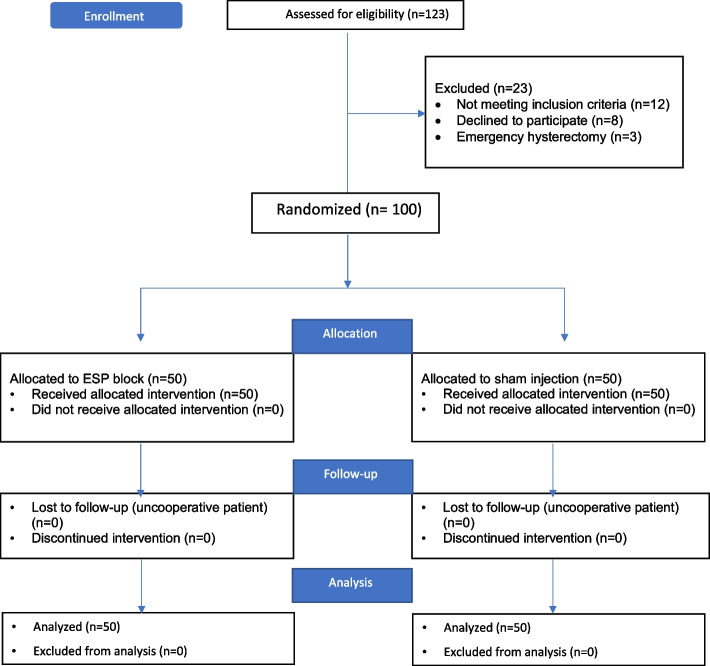
Consort flow diagram of the study population

Of the 123 patients evaluated for eligibility, 12 did not meet the inclusion criteria, eight refused to participate, and three had an emergency hysterectomy. Data for 100 patients were analyzed (Fig. 1).

There was no significant difference between groups regarding the patients' demographic characteristics (Table 1).

**Table 1:** The patients' demographic characteristics and operative data

	**The ESPB groups**	**The control group**
Sample size,n	50	50
Mean age (SD) in (years)	51) 9 (	53 (9)
Mean weight (SD) in( kg)	75 (13 (	77 (12)
ASA, n (%)		
1	35 (70)	30 (60)
11	15 (30)	20 (40)
the mean (SBP) at the beginning of the operation in mmHg	131.3 (12.4)	134.8 (11.4)
the mean (SBP) at the end of the operation in mmHg	128.3 (12.04)	142.3 (3.7)

*Abbreviations*: *SD* Standard deviation, *n* number, *ASA* American society of anesthesiologists, *ESPB* Erector spinae plane block, *SBP* Systolic blood pressure

There was no significant difference between groups regarding systolic blood pressure during the operation (tthe mean systolic blood pressure at the beginning was 131.3 (12.4) mmHg in the ESPB vs. 134.8 (11.4) mmHg in the control group; *p* = 0.3). However, at the end of the process, 128.3 (12.04) mmHg in the ESPB vs. 142.3 (3.7) mmHg in the control group was *p* = 0.08 (Table 1).

Table 2 shows that the mean (SD) intraoperative fentanyl consumption was significantly lower in the ESPB group than in the control group (82.9 (27.4) g vs. 148.5 (44.8) g, with a 95% CI = -80.3 to -50.8; *p* 0.001). Likewise, mean (SD) postoperative fentanyl consumption was significantly lower in the ESPB group than in the control group (442.4 (17.8) g vs. 477.9 (10.4) g, with a 95% CI = -41.3 to -29.7; *p* 0.001). On the other hand, there is no statistically significant difference between the two study groups regarding sevoflurane consumption (89.2 (19.5) ml vs. 92.4 (15.3) ml, with a 95% CI = -10.1 to 3.8; *p* 0.4).

**Table 2:** Fentanyl and sevoflurane consumption

	**The ESPB group**	**The control group**	**Mean difference 95% CI**	***P*-value**
Sample size,n	50	50		
Mean intraoperative fentanyl consumption (SD) in (µg)	82.9 (27.4)	148.5 (44.8)	-80.3 to -50.8	< 0.001
Mean postoperative fentanyl consumption (SD) in (µg)	442.4 (17.8)	477.9 (10.4)	-41.3 to -29.7	< 0.001
Sevoflurane consumption (ml)	89.2 (19.5)	92.4 (15.3)	-10.1 to 3.8	0.4

*Abbreviations:* *SD* Standard deviation, *n* number, *SPB* Erector spinae plane block

**Fig. 2:**
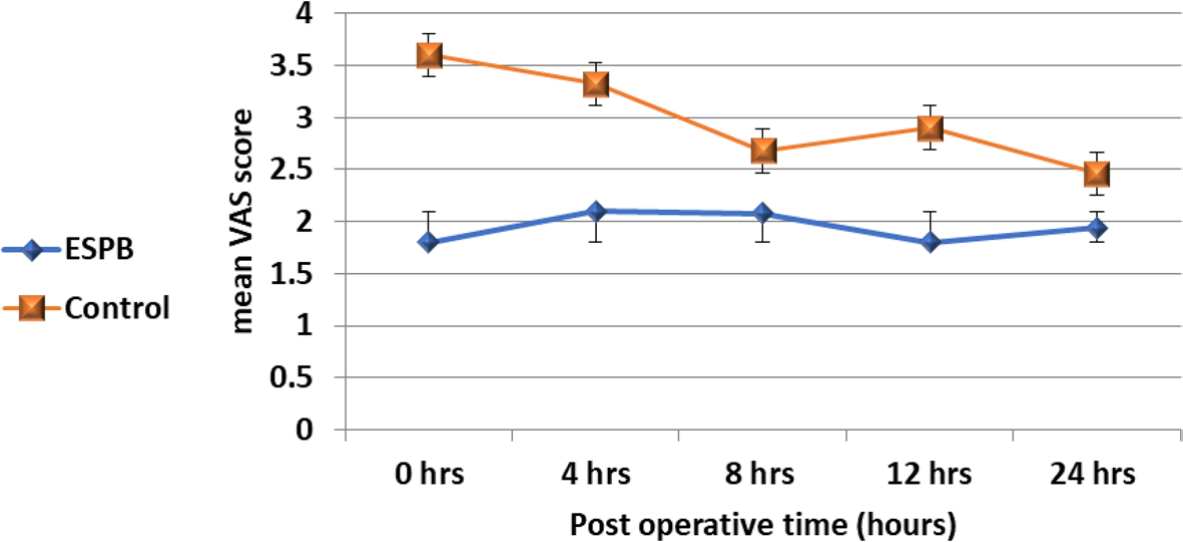
The VAS score at rest

Figure 2 shows that during the post-operative period (0–24 h), VAS scores at rest were, on average, 1.03 units lower in the ESPB group (estimate = -1.03, 95% CI = -1.16-(-0.86), t = -14.9, *p*-value 0.001).

**Fig. 3:**
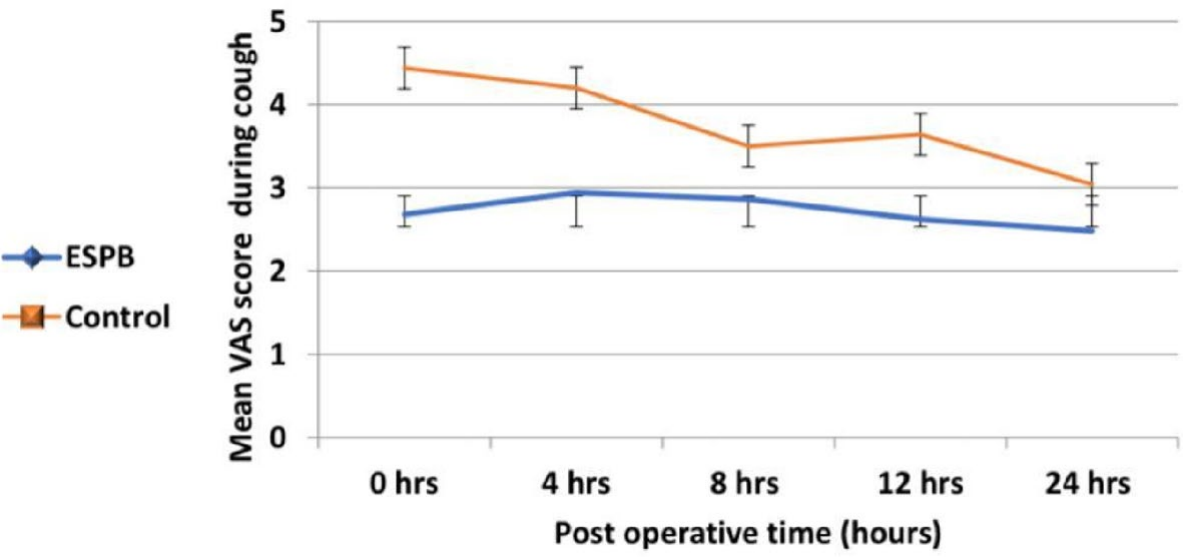
The VAS score during cough

Figure 3 shows that during the post-operative period (0–24 h), VAS scores during cough were, on average, 1.07 units lower in the ESPB group (estimate = -1.07, 95% CI = -1.21-(-0.93), t = -14.8, *p*-value 0.001).

No complications were observed among the groups.

## Discussion

When comparing the intraoperative and postoperative doses of fentanyl in our clinical experiment, there is a statistically significant difference between the two groups, with the ESPB group consuming less of the fentanyl. However, there is no statistically significant difference between the groups in sevoflurane consumption.

The effectiveness of the ultrasound-guided erector spinae block versus the subcostal approach to the transversus abdominis plane block in obese patients undergoing sleeve gastrectomy was compared in Abdelhamid and colleagues' [13], prospective, randomized, double-blinded controlled study, which was conducted in 2020. They found that the need of intraoperative and postoperative opioids was decreased with ultrasound-guided single-shot T9 ESPB in contrast to the subcostal approach TAP block and the control group.

Elyazed and colleagues' arguments were supported by a recent study as well [14]. In 2019 (T6-T9), researchers carried out a prospective randomized controlled study (TRC) to examine the analgesic efficacy of bilateral ultrasound-guided ESPB in patients having open midline epigastric hernia repair. They claimed that ultrasound-guided bilateral ESPB reduced the need for both intraoperative fentanyl and postoperative rescue analgesia. Only 4 patients in the ESPB group needed intraoperative fentanyl, compared to 27 patients in the control group. The median (quartile) intraoperative fentanyl intake in the ESPB group was also significantly lower than in the control group.

According to the statistical analysis of the VAS scores in the current study, there was a statistically significant difference between the groups' follow-up from the immediate postoperative period until 24 h, with the ESPB group having a lower mean score.

Kamel et al. [15] in their study of bilateral ultrasound-guided erector spinae planeblock versus transversus abdominis plane block on postoperative analgesia after total abdominal hysterectomy, 48 women were randomly allocated into two equal groups: The erector spinae group received bilateral ultrasound-guided ESPB with 20 mL of bupivacaine 0.375% plus 5 ug/mL adrenaline (1:200,000) on each side at the level of T9, and the transversus abdominis group received bilateral ultrasound-guided TAP block with the same volume of bupivacaine plus adrenaline. They concluded that bilateral ultrasound-guided EP block provides more potent and longer postoperative analgesia with less morphine consumption than transversus abdominis block after the open total abdominal hysterectomy.

Altinpulluk et al. [7], in their trial, which included ten patients starting from lower thoracic levels (T9), provided effective postoperative analgesia in open abdominal hysterectomies.

Prasad and colleagues [16] in their clinical trial, Peripheral nerve stimulator-guided erector spinae plane block for post-operative analgesia after total abdominal hysterectomies, concluded that the ESPB is effective in improving pain in females undergoing total abdominal hysterectomies.

Hamed and colleagues [6] conducted a randomized controlled experiment in 2019 to evaluate the effectiveness of bilateral ESPB on postoperative analgesia in females undergoing total abdominal hysterectomy (TAH) under general anesthesia. This study supported their findings. They claimed that in patients receiving TAH, bilateral ESPB significantly reduced postoperative fentanyl usage and provided acceptable postoperative analgesia. Additionally, the control group's total fentanyl intake in the first 24 h was considerably higher than the ESPB group's (*P* = 0.003; 48,520.39 mcg vs. 44,567.49 mcg, respectively), and the control group's VAS for pain was significantly higher in the first 12 h postoperatively.

The current study also concurred with Shukla and colleagues [17]; in 2022, they assessed and carried out a randomized comparative study between bilateral ESPB and transversus abdominis plane block under ultrasound guidance for postoperative analgesia after total abdominal hysterectomy. They came to the conclusion that bilateral ultrasound-guided ESPB causes appropriate analgesia and a reduction in the need for analgesia with less tramadol consumption compared to ultrasound-guided transversus abdominal plane block in patients after total abdominal hysterectomy.

Table 3 compares different studies related to the use of ESPB in open hysterectomy.

**Table 3:** Compares different studies related to the use of ESPB in open hysterectomy

References	Group	Number of patients	Anesthetic and concentration	Conclusions
Kamel et al. [15]	bilateral ultrasound-guided erector spinae plane block versus transversus abdominis plane block	48	20 mL of bupivacaine 0.375% plus 5 ug/mL adrenaline (1:200,000) in each side at the level of T9	bilateral ultrasound-guided esp block provides more potent and longer postoperative analgesia with less morphine consumption than transversus abdominis block after the open total abdominal hysterectomy
Altinpulluk et al. [7]	bilateral ultrasound-guided erector spinae plane block	10	15 ml of 0.25% bupivacaine was injected as LA on each side at the level of T9	Bilateral postoperative ultrasound-guided ESP block can result in a good sensory blockade and visceral analgesia
Prasad and colleagues [16]	Peripheral nerve stimulator guided erector spinae plane block	60	20 mL of 0.375% ropivacaine at the level of T10	the ESPB is effective in improving pain
Hamed and colleagues [6]	bilateral ESPB group and control group	60	20 ml of 0.5% bupivacaine was injected as LA on each side at the level of T9	bilateral ESPB significantly reduced postoperative fentanyl consumption and provided acceptable postoperative analgesia
Shukla and colleagues [17]	bilateral ESPB and Transversus Abdominis Plane Block	30	mixing 20 ml of 0.5% bupivacaine plus 10 ml of 2% lignocaine and 1 ml (50mcg) of fentanyl and 9 ml of normal saline forming total 40 ml of which 20 ml was injected on each side	bilateral ultrasound-guided ESPB causes appropriate analgesia and a reduction in the need for analgesia with less tramadol consumption compared to ultrasound-guided Transversus Abdominis Plane block

### Limitations

All of the clinical study participants were Egyptian, which restricted the data's applicability to people of other races. Fentanyl consumption and VAS scores were only evaluated during the trial's 24-h period.

## Conclusion

To reduce intraoperative fentanyl use and improve postoperative pain management in patients having open total abdominal hysterectomy under general anesthesia, bilateral ESPB is a safe, efficient, minimally invasive adjuvant technique.

## Data Availability

The datasets used and analyzed during the current study are available from the corresponding author upon reasonable request.

## Abbreviations

ASA: The American Society of Anesthesiologists
BIS: Bispectral Index
CI: Confidence Interval
ESPB: Erector spinae plane block
HR: Heart Rate
IQR: Interquartile Range
NRS: Numeric Rating Scale
PACU: Post Anesthesia Care Unit
PCA: Patient-Controlled Analgesia
SBP: Systolic blood pressure
SD: Standard deviation
TAH: Total abdominal hysterectomy
